# Antimicrobial stewardship: a qualitative study of the development of national guidelines for antibiotic use in hospitals

**DOI:** 10.1186/s12913-017-2683-4

**Published:** 2017-11-21

**Authors:** Eli Feiring, Anne Berit Walter

**Affiliations:** 1 Department of Health Management and Health Economics, University of Oslo, Box 1089 Blindern, 0317 Oslo, PO Norway; 2 Department of Life Sciences and Health, Oslo and Akershus University College of Applied Sciences, Box 4 St Olavs Plass, 0130 Oslo, PO Norway

**Keywords:** Antimicrobial resistance, Qualitative study, Implementation, Guidelines, Evidence-based public health, Accountability for reasonableness

## Abstract

**Background:**

As effective antibiotics are becoming a scarce resource, governmental regulation is needed to promote responsible use. Implementation of antibiotic stewardship and practice guidelines in health care facilities seems to be crucial to this effort. Empirical studies suggest, however, that guidelines have limited influence on health professionals’ behavior and practice. Barriers and facilitators to guideline implementability are much studied, but little attention has been given to health professionals’ perceptions of normative acceptability of guidelines as a condition for compliance. The aim of the present study was first, to examine if and how aspects potentially promoting acceptability and compliance among clinical target users were addressed during development of Norwegian national guidelines for antibiotic use in hospitals and second, to identify procedural characteristics of the development process that were perceived by target users to yield legitimate guidelines.

**Methods:**

Qualitative deductive thematic analysis was used. A theoretical framework inspired by the AGREE II Instrument and the Accountability for reasonableness framework assisted data gathering and interpretation. Archival data was collected and used to detail the guideline development process. Semi-structured, in-depth interviews with eight clinicians with extensive knowledge of the guidelines were carried out.

**Results:**

Guideline development was characterized by i) broad agreement about scope and purpose, ii) broad involvement of stakeholders in the development process, iii) use of systematic methods to search for and apply evidence, iv) easily identifiable and specific recommendations, v) provision of tools on how to put recommendations into practice, and vi) editorial independence. Several procedural characteristics were perceived by the interviewees as promoting guideline legitimacy; i) diverse perspectives systematically involved in the process, ii) accessibility and transparency of the rationales for decision making, iii) opportunities for appeals and reconsiderations, and iv) regulative authority.

**Conclusions:**

This study provides insights as to how guidelines that are intended to promote responsible use of antibiotics in hospitals can be carefully developed to facilitate perceptions of relevance, transparency, and authority by health professionals.

## Background

Antibiotics were introduced over 75 years ago and are now indispensable in all health systems. There is, however, a loss of antibiotic effectiveness due to a rapid evolution of antimicrobial resistance (AMR) [1–3]. The causes of AMR are complex. Unnecessary use of antibiotics and inappropriate use of broad-spectrum antibiotics are important factors. At the same time, few new antibiotics have become available as a result of a market failure of antibiotic development. The consequences are severe. AMR results in higher rates of morbidity and mortality in patients with resistant infections and increased healthcare costs due to prolonged hospital stays and more expensive drugs. It also leads to inability to make use of interventions that rely on effective antibiotics, including surgery, cancer chemotherapy, transplantation medicine and care of premature infants and the critically ill. The effects of AMR extend beyond the individual as resistant microbes are transmitted among humans and between humans and the environment. Not only is individual safety at risk, but public health is threatened.

To reverse antibiotics’ decline, legislative action, funding, and public policy strategies are needed. Among the different policy activities available is the regulation and promotion of responsible use of medicines to ensure proper patient care [1]. Antimicrobial management or stewardship should be developed to “improve and measure the appropriate use of antimicrobials by promoting the selection of the optimal antimicrobial drug regimen, dose, duration of therapy, and route of administration” [2]. A significant variation in relation to organizational structures and interventions of antimicrobial steward programmes exist and a number of strategies, policies and tools may be used to optimize antibiotic use [4]. Clinical practice guidelines seem to be central to this effort, especially in settings where prescribers act as gatekeepers to antibiotic access.

Yet, practice guidelines become irrelevant if they are not known, adopted and used by the health professionals they target. Research has documented that guidelines in general have limited influence on health professionals’ behavior and practice [5, 6]. A broad range of barriers and facilitators to implementation of guidelines have been identified in the literature [7]. However, little attention has been given to health professionals’ views about justifiability and acceptability of guidelines as a condition for compliance. This is unfortunate because it is commonly presumed that public governance will be respected only if is it seen as fair and legitimate [7, 8]. In the present study, we wanted to better understand how the process by which health authorities develop evidence-based guidelines can be enhanced to promote regulation of practice that clinical target users find legitimate. The specific aim was twofold. First, we wanted to examine if and how aspects potentially promoting acceptability and compliance among target users were addressed during development of Norwegian national guidelines for antibiotic use in hospitals. Second, we wanted to identify procedural characteristics of the development process that were perceived by target users to yield legitimate guidelines.

It is important to note that we did not aim to assess the quality of the guidelines in numerical terms or to evaluate the clinical validity of the recommendations themselves. The Norwegian national guidelines for the use of antibiotics in hospitals can be accessed at https://helsedirektoratet.no/retningslinjer/antibiotika-i-sykehus.

Before presenting the methods and findings, we give a short summary of the formal process of guideline development in Norway.

### Clinical practice guidelines development in Norway

The Norwegian Directorate of Health is responsible for national clinical guideline development in Norway. Development of guidelines follows a standard model that aims at independence, high reliability, transparency in process and inclusiveness by various stakeholders, rigor in methodology, and systematic use of evidence [9]. Suggestions for guideline topics come from health care professionals, patient organizations, the Ministry of Health and Care Services or the Directorate of Health. The Directorate of Health selects topics of priority on the basis of several criteria (such as burden of disease, degree of variation in clinical practice and outcomes across the nation, the impact on resources and the importance of policy). When a particular topic is decided on, a formalized development process begins. A working-group from the Directorate of Health is supported by technical experts in systematic reviewing, health economics and information science. Various stakeholders, including clinicians, providers and patients, are invited to be part of the process. Recommendations should be based on the best available evidence for the appropriate treatment of the patient. It is presumed that the GRADE methodology (Grading of Recommendations, Assessment, Development and Evaluation), which is an international collaborative initiative to increase quality of guideline development by grading the strength of recommendations [10], is used to summarize and assess clinical effectiveness, and organizational and economic consequences are assessed. The guideline draft is internally reviewed and stakeholders are invited to submit comments during a hearing period. A plan for implementation and revision is developed.

## Methods

### Study design

The present study was designed as a qualitative case study. A case study makes it possible to study a single unit in detail, linking general knowledge to empirical knowledge of the more specific mechanisms in an individual case [11]. This study’s data was collected by means of systematic reading of archival data and qualitative in-depth expert interviews. Data was interpreted within a deductive thematic analysis framework [12]. We wanted theory-before-research to assist design research questions, guide the selection of relevant data, aid in defining an appropriate description and interpretation of data, and eventually help move beyond specific insights from the single case we set out to study [13, 14].

### Setting

Norway is characterized by high per capita income and an egalitarian ideological orientation. Health care is need-based, universal, and tax-financed. Specialist care is organized in four health regions. There is no second private tier. Thus, the patients’ ability to pay does not enter the decision-making process concerning use of antibiotics in hospitals.

Norway has a relatively low rate of AMR but the rate is increasing, notably for methicillin-resistant *Staphylococcus aureus* (MRSA), vancomycin-resistant bowel bacteria and extended spectrum beta-lactamase-resistant bacteria (ESBL) [15]. Resistance is monitored in several ways, for example by the Norwegian Resistance Monitoring and Surveillance Programme for Antimicrobial Resistance and the Norwegian Surveillance System for Communicable Diseases. Center for Use of Antibiotics in Primary Care and Norwegian Advisory Unit on Antibiotic Use in Hospitals are established to promote rational use of antibiotics, in addition to the Norwegian Advisory Unit on Detection of Antimicrobial Resistance.

Hospitals are responsible for approximately 9% of total antibiotic consumption [16]. There are, however, significant variations in use of antibiotics between hospitals. In 2014, less than 50% of the hospitals had an antibiotic strategy [17].

### Analytical framework

Clinical practice guidelines have evolved as part of the evidence-based approach to medicine and are defined as *systematically developed statements to assist practitioners and patient decisions about appropriate healthcare for specific clinical circumstances* [18]. Guidelines have been used to support decision-making in the clinical setting and to contribute to improvement of quality of healthcare since the 1980ies. Over time, international collaborative initiatives have been taken to increase quality of guideline development. In 2003, the AGREE instrument for assessing the quality of guidelines was published as a result of an international collaboration (Appraisal of Guidelines, Research and Evaluation Collaboration) [19]. The instrument was refined in 2009, now called AGREE II, to assess the quality of guidelines across the spectrum of health, provide directions on development, and guide what information ought to be reported in the guidelines [20]. AGREE II groups 23 items in six overall domains that are thought to reflect significant aspects of the guideline development at different steps in the lifecycle of guideline management, and to have implications for implementation. The six domains and their corresponding items are: Scope and Purpose (objectives, health questions, target population); Stakeholder Involvement (health professionals, target population, target users); Development (methods, evidence selection and strengths, recommendation formulation, benefits/risks, link between recommendations and evidence, reviews, update procedure); Presentation (recommendation specificity, clarity, and identifiability); Applicability (tools/advice for use, facilitators/barriers to implementation, resource implications, auditing); and Editorial Independence (funding body, interests of development group).

AGREE II can be used to quantitatively evaluate the methodological rigor of a practice guideline, as each of the items can be rated on a point scale [18]. Recent examples of quantitative application include [21, 22]. For the purpose of the present study, we used the domains and items defined in AGREE II to develop a framework for qualitative analysis. Thus, the domains and items described were used to structure the gathering and presentation of data.

Guidelines for responsible use of antibiotics are essentially limit-setting. Informed by quality improvement literature, we assumed that target users‘views of guideline acceptability were associated with their views about the reasonableness of the development process [7]. The AGREE II instrument does not, however, address normative perceptions of acceptance, justifiability, and legitimacy. To inform the analysis and interpret notions of legitimate governance, we made use of the normative-political framework of Accountability for reasonableness developed by Daniels and Sabin [8, 23]. The framework is a characterization of general conditions a process for setting priorities among health needs must meet if it is to yield outcomes that are perceived as fair and legitimate. These conditions relate to questions of how the process is organized, about who is involved, and about the rationale for decisions.

According to the framework, fair allocation of resources requires that health care decision-makers are accountable for the reasonableness of their regulative behavior. This means that decisions about setting limits must rest on evidence, reasons and principles that all stakeholders consider relevant (Relevance Condition). Further, decisions must be publicly accessible and transparent and there must be opportunities for revision and improvement of policies in light of new evidence or arguments (Publicity and Revision Conditions). The decision-making process should be regulated to ensure that these conditions are met (Regulative Condition).

For the purpose of this study, we assumed that normative conditions such as relevance, transparency, revisability, and regulative authority, are important to the individual clinician’s assessment of regulations that imply restrictions of clinical autonomy for societal purpose.

**Fig. 1:**
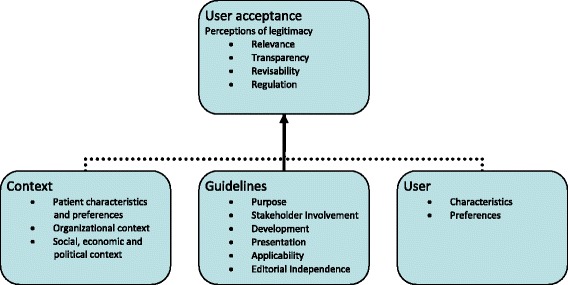
Guideline acceptance: Analytical framework

Figure 1 describes the analytical framework of the present study. It is suggested that the development of the guideline process ought to be designed carefully to promote acceptance among target users. This may imply (but is not limited to) that a range of factors needs to be considered by the governmental agency guideline developers, such as: How to reach a broad agreement about the purpose of the guideline; How to minimize conflict of interest among guideline developers; How to deal with an expected heterogeneity of patient characteristics and preferences; How to ensure adequacy of evidence base that support the recommendations; How to develop a revision and implementation strategy that is feasible; How to ensure public accessibility regarding decisions and their rationale. In addition to characteristics of the guideline development process, other factors (the individual health professional, the patient, the organization, and the social, economical and political context) are important for implementation [7]. These factors were, however, not a central focus of the present study.

### Archival data

Archival data was gathered to aid the understanding and interpretation of the rationale behind the development of the national guidelines for antibiotic use in hospitals, the development process and the relevant political context. Data was collected from the Norwegian Directorate of Health (e.g. formal steering documents concerning the general guideline development process, the mandate of the working group), from official documents (relevant laws, white and green papers, official Norwegian reports, propositions to the Parliament and strategy documents) and through the National Advisory Unit for Antibiotic Use in Hospitals and Norwegian Institute of Public Health. All documents were publicly available and did not require permission to access.

### Interview data: Participants

Eight clinicians (seven hospital doctors and one doctor from primary health care) working at different health enterprises from different parts of Norway were strategically selected and interviewed in 2014. Five of the interviewees had been key participants in the specific guideline development process and had extensive knowledge of the process. The other interviewees were hospital doctors that knew the old and new guidelines well. All hospital doctors were considered as target users of the guideline. The sample was partly identified ex ante according to the position and experience the interviewees held, and partly by chain-referral, where the initial set of interviewees was supplied with clinicians nominated by those already interviewed.

A theoretically driven approach was used to aid the data gathering and analysis. As such, we did not aim to explore a full range of themes grounded in the data itself (saturation). We wanted to study how clinicians framed their views about the guideline development process and why they held these views. The number of interviewees was small but was, in combination with archival data, determined to be adequate to answer our research question.

### Interviews

Interview data was collected by means of qualitative in-depth expert interviews. The interviews were semi-structured and were based on an interview guide which was thematically organized according to the six domains of the AGREE II instrument. Open-ended responses were allowed to elicit personal experiences and perspectives and to uncover as much information as possible.

All interviews were conducted in Norwegian, audio-taped and transcribed verbatim. The transcribed data was analyzed in collaboration between the two investigators. Data was read through to get a sense of the overall content, content referring to the main AGREE II themes was identified, and text units with statements were entered into tables, and merged to new themes referring to normative conditions (relevance, transparency, revisiability, and regulative authority).

Each interviewee was given the opportunity to review the relevant transcript to identify any corrections. The most relevant parts were translated into English.

## Results

Our findings are reported below, organized on the basis of AGREE II domains (Scope and Purpose; Stakeholder Involvement; Development; Presentation; Applicability; and Editorial Independence). Table 1 summarizes the findings and quotes from interviewees are included for illustration. Normative considerations that were addressed by the interviewees are here categorized with reference to the conditions for fair and legitimate decisions formulated by the Accountability for reasonableness framework (Relevance; Transparency; Revisability; Regulative authority).

**Table 1:** Summary of results

Domains (cf. AGREE II)	Archival data – examples of findings	Interview data – examples of quotas	Normative conditions addressed
Scope and Purpose	Guideline rationale: Growing AMR Guideline purpose: Responsible use of antibiotics in hospitals Guideline target: Health professionals in hospital settings Guideline initiative: Political and health professional Development responsibility: Norwegian Directorate of Health	“I don’t think there has been some great discussion about whether (antibiotic) guidelines are needed. There are always some who think it’s nonsense, but we know that summarizing what’s out there is good, so that one doesn’t have an unfortunate practice compared to the evidence-base”. “I think everyone needs the guidelines, because the specialists are not necessarily on duty and they are not always available (...) and even to us (the specialists), well it is a large area, having the guidelines as a pillar, not least, it is important”. “Why should we change practice when the local guideline works well? The national (guideline) is important but I wouldn’t accept everything at face value, to put it simple”.	Guideline relevance
Stakeholder Involvement	Development process: Project organizing - Steering group - Guideline development group - Expert groups Stakeholder representation: - Health authorities - Norwegian Medicine Agency - Norwegian Institute of Public Health - National Advisory Unit for Antibiotic Use in Hospitals - Antibiotic Centre for Primary Care - Norwegian Federation of Organizations of Disabled People - a.o. Expert involvement: >80 health professionals	“I believe that the process was very good because the guidelines were anchored in a wide academic environment, that is, in several disciplines. These are guidelines that all hospital doctors (….) should be aware of. And it was important that representatives, influential representatives that is, from each discipline were included, representatives appointed by the different specialist associations.” “When people are a part of the process they experience a sense of ownership. And these people are influential people at the different hospitals”. “When so many people from infectious disease medicine and micro-biology are involved in the development of the guidelines, they (the guidelines) get a totally different impact and credibility (...). If people disagree at least they know that many and mostly well trained people have approved the outcome.” “I think that there is a greater risk that one can come up with guidelines that are not being used if you don’t do it this way (…). It is the spirit of *dugnad*”.	Relevance of evidence, reasons and their foundations Transparency of decision-making process
Rigor of Development	GRADE methodology for some recommendations Systematic literature reviews using PICO Various data sources Recommendations based on specific criteria Formalized strategy for implementation and update Update responsible: National Advisory Unit for Antibiotic Use in Hospitals	“The new guidelines have a clear advantage in that they to a greater extent are evidence-based, with references, and (are the outcome of) a transparent process. The authority of the new guidelines is great, I think”. “It’s definitely a need to have a “live” guideline”. “It is the fresh produce really. Infectious disease medicine changes quickly”.	Relevance of evidence, reasons and their foundations Transparency of decision-making process Revisability of guidelines
Clarity of presentation	Recommendations presented as strongly or conditionally recommended Tables summary of recommendations	“I don’t think it’s good if there are changes that make our work more cumbersome, but changes that make the work easier (…) then I’m all in”.	Transparency of recommendations
Applicability	Electronic publication Standard and short version	“In everyday life you want everything to be easily available (…). I want a leaflet to have in my pocket”. “Everyone has a smart phone now (…). And with an app, well that would be great”.	
Editorial independence	Guideline developed by the Norwegian Directorate of Health	“If there is something that is published on the Web by the Norwegian Directorate of Health, you think it’s updated, the latest, it is that which applies”.	Guideline relevance

### Scope and purpose

Archival data show that a national plan against antibiotic resistance was developed as a result of a political initiative by five Ministries (Labor and Social Affairs, Agriculture and Food, Fishery, Climate and Environment, and Health and Care Services) in 2008 [24]. The plan identified a need for a coherent approach to the development and maintenance of guidelines for rational use of antibiotics in hospitals. It also identified lack of knowledge regarding the content and actual use of local and regional developed guidelines and it was suggested that guidelines should be updated locally with the support of national and regional health authorities. Two years later, an initiative to develop national guidelines for antibiotic use in hospitals was taken by a group of health professionals.

The Directorate of Health supported the initiative and was assigned the responsibility for the development process. The new national guidelines for use of antibiotics in hospitals were published in 2013 [25]. The purpose of the guidelines was to foster responsible use of antibiotics, providing *the most effective antibiotic against the pathogenic bacterium with the least possible impact on the body’s normal bacterial flora and the least amount of resistance development*. The rationale was explained by stating the fact that antibiotics are a limited resource and that overuse and wrong use is followed by growing resistance, increasing costs and negative side effects.

Interview data suggest that the interviewees perceived the governmental decision to develop national guidelines for the use of antibiotics in hospitals to have broad professional support. They pointed out that local and regional guidelines were already in use and that the new national guidelines complied with existing opinions and routines of the target group. In their opinion, the new guidelines did not differ much from the previous guidelines they were already used to. One remarked that this fact undercut the need for new national guidelines. Others stressed that up-to-date guidelines were welcomed as an aid for decision-making. All interviewees regarded the overall aim of the guideline development, i.e. responsible use of antibiotic in hospitals, as relevant and appropriate.

### Stakeholder involvement

Archival data state that the development process was organized as a project with a given mandate, specifying responsibilities and tasks, and plans for implantation [25]. The process was organized in several groups. A guideline development group involved representatives from various organizations. More than 80 health professionals from different relevant disciplines participated in various expert groups. A steering group with representatives from the Directorate of Health and the guideline development group coordinated the process. The work was organized with meetings over a two-year period. Expertise in fields like health economics and scientific methods was made available. The final draft was given a rather short period for hearing (a little more than a month). The many comments that were received was discussed and incorporated into the final document.

The development process aimed at involving a variety of stakeholders from various parts and levels of the health care sector, from a wide range of disciplines, as well as from different geographical parts of the country. However, only two patient representatives participated in the process.

The broad representation of health professionals was perceived as a vital facilitator for guideline acceptance by the interviewees. They emphasized that a variety of perspectives was brought to the table and assessed and they regarded this feature of the process as a major strength. It was pointed out that involving a range of different disciplines was likely to increase guideline relevance and the feeling of ownership among target users. Several described the process as a *dugnad*. The Norwegian term *dugnad* refers to unpaid orchestrated community-work where people get together to help each other carry out tasks that is difficult on an individual basis. The “spirit of *dugnad*” was thought to facilitate transparency in the development process. Further, the involvement of local opinion leaders was emphasized as potentially important to improve guidance acceptance.

### Rigor of development

The GRADE methodology was used for some, but not many, of the recommendations [25]. Systematic literature reviews were made with the use of the PICO instrument (Population, Intervention, Comparison, and Outcome), but the process also included unsystematic literature review. Eventually, the data basis for the recommendations consisted of meta-reviews, systematical reviews, guidelines, high quality international studies, and some local studies from the Scandinavian context. These were included also when quality was low. A strategy for implementation and update was developed and the National Advisory Unit for Antibiotic Use in Hospitals was assigned responsibility to implement the national guidelines. Some of the activities prescribed in the strategy were targeted at applicability, such as making the guidelines integrated in the electronic patient journal and making a short paper version and apps for smart telephones. Other activities targeted organizational and pedagogical challenges, such as providing information about antibiotic resistance, involving leadership at all levels of decision-making in the implementation of the guidelines, and contributing to surveillance of antibiotic use and resistance. However, the guidelines did not entail a description of a process of assessing when guidelines should be updated.

Interview data suggest that the perceived rigor and transparency of the development process was seen as a characteristic adding to the authority of the guidelines. The rapidly evolving field of infectious disease medicine was pointed out by the interviewees as a challenge. They were concerned about the needs for evidence-based up-date and revision of the guidelines. As such, the guidelines were seen as a summary of research findings and current knowledge. The possibility for revision and improvement was regarded as an essential condition for acceptability.

Archival data show that the guideline developers expressed awareness of the fact that there was lack of evidence for appropriate grading approaches and that GRADE was used to a lesser degree. This issue was not addressed by the interviewees.

### Presentation and applicability

The guidelines were published electronically, in a standard and a short version, for professional use [25]. The different recommendations were presented as *strongly* or *conditionally* recommended, depending on (i) the quality of evidence, (ii) the balance between patient needs and societal risk of resistance, (iii) patient preferences, and (iv) cost-effectiveness. The recommendations were summarized in tables. The standard version included general information about antibiotic resistance, aims and purpose of the guidelines, descriptions of the development process, the scientific methods used, resource implications and changes.

Interview data suggest that accessibility and applicability was a main concern for the interviewees. Some found the standard full-length text too demanding. In addition, it was pointed out that the electronic solutions at the hospitals were sub-optimal, making the guidelines less applicable in the relevant clinical setting.

### Editorial independence

Guideline development processes may result in recommendations that are criticized for strengthen some professional groups at the cost of others. This was not a finding in our data. The interviewees placed great trust in the editorial independence of the guidelines. The Directorate of Health, which is responsible for national guideline development and subsequently for guideline recommendations, was perceived as a guarantor of neutrality between competing professional interests.

## Discussion

The development of AMP is described by the World Health Organization as a worldwide public health crisis [1]. Countries are called upon to address a range of specific health aspects to contain AMR. The Lancet Infectious Diseases Commission has reported that countries that have implemented comprehensive national strategies to control resistance have been most successful [3]. Organized antimicrobial stewardship activities in hospitals vary considerably across the world [4]. In Norway, national stewardship standards are only recently initiated. This study analyzes how national guidelines for the use of antibiotic in hospitals were developed as a tool for improving responsible clinical practice. Archival and interview data show that the six domains of guideline quality described by AGREE II were addressed during the development process. All four features of the Accountability for reasonableness framework were identified in the data.

First, broad agreement on purpose can contribute to raised motivation and organizational readiness among target users [26, 27]. In this study, we found that health authorities decided to develop national guidelines to meet the challenges of growing antimicrobial resistance and variations in antibiotic use between hospitals. The initiative to develop national guidelines was taken by health professionals. Thus, there existed an initial mutual disposition to deciding on national guidelines. This finding of archival data was supported by interview data and was recognized by the interviewees to add to perceptions of guideline relevance.

Second, the composition of the development groups is shown to be important for the validity and acceptance of a guideline [7]. Including a range of stakeholders in the developing process may increase the likelihood that a broader pool of relevant reasons will be aired and eventually considered. A broad representation may convey the transparency the process requires to be perceived as fair. Further, the rationales for decisions should be public to facilitate transparency and openness. It is important that target users understand how and why limits are being set and are given an opportunity to participate in the development process. Our data show how the guideline development process included persons from all relevant professional groups, health authorities at different levels, and patient representation from organizations. Further, the data indicate that the interviewees found the broad representation to be of vital importance for guideline acceptability.

Third, a structured and rigorous process, with methodological support for collection and evaluation of scientific evidence, is essential for credibility [7]. Systematic review has become a cornerstone of the evidence-based medicine and is developed as a specific methodology for searching for, appraising and synthesizing research findings with the aim to provide rigor and transparency of process. Yet, the systematic review of evidence is perhaps the most costly and time-consuming component in the guideline development process because of the necessity to collect all the relevant evidence. In this study, we found that a mix of “hard” and “soft” forms of evidence was used. In addition to some GRADE evaluations, sources of evidence included context-dependent studies with local applicability. The interview data indicate a tendency to consider the guidelines as useful decision-aid as long as they are easily accessible and applicable. This finding is in accordance with a recent Norwegian study that reported that the doctors’ attitudes towards the guidelines negatively corresponded with level of experience [28].

Fourth, should new evidence come to light, reconsiderations must be made. Our data illustrate how key recommendations were easily identifiable and provided different tools for practice. Electronic resources and different guideline formats were used. There was a public comment period and a formal procedure for updating guidelines was provided. The opportunities for revision and improvement of revisions were regarded by the interviewees to be crucial for guideline acceptability.

The Directorate of Health is the government agency responsible for the development of national guidelines in Norway and the process was designed to follow the Directorate’s model for guideline development. As such, the formal condition of enforcement of the substantive conditions (relevance, publicity and revisability) was met.

The Accountability for reasonableness framework underscores how giving reasons is a way to achieve acceptance and compliance from stakeholders who have diverse perspectives on the limit-setting decision under discussion [8, 23]. The reasons offered must be ones that all can accept as relevant and appropriate. Further, decisions and their rationales must be publicly accessible and revisable. Our data show how the development process was designed to enhance acceptability by the target users. Further, data indicate how procedural characteristics of the guideline development process were perceived by the interviewees to promote relevance, transparency, revisability and authority.

### Implications and further research

Guidelines for the use of antibiotics may encounter special challenges in relation to implementation because results of responsible antibiotic use that have effects in large and future populations, may be thought by the individual health professional and the patient to be counter-productive at the individual level. In addition, the immediate risk for the present patient must be balanced against the uncertain effect of AMR in the distant future. AMR thus becomes a “theoretical” or distant problem compared with the potentially severe consequences for the individual patient of not prescribing antibiotics [29, 30]. A study from Sweden found that while the doctors were aware of the existence of AMR, they expressed an overarching concern for the present patient and did not seem to be prepared to change to restrictive prescribing if they did not have a special interest in infectious disease management or had support of colleagues, such as an infectious disease specialist [31]. This issue was not addressed in our study.

A decision to protect a population against one health risk, such as antimicrobial resistance, as opposed to another, such as bacterial infections, will advantage some and disadvantage others. Further studies should address decision-makers’ and health professionals’ perceptions of the underlying ethical dilemma of how to weigh considerations of the risk of the present patient against societal risk of antimicrobial resistance. The consequences of antibiotic use will ultimately affect a large number of people (as well as a range of different sectors and policy areas) and the evidence base may be difficult to define, as well as to interpret. Further, reasons that may be compelling to the decision-maker will not always seem relevant to those affected by the decision. Patient and public involvement may become more important as the decisions to protect the population against the risk of antimicrobial resistance as opposed to the risk of bacterial infections require broad acceptance of antibiotics rationing.

These observations point to a fundamental challenge for antimicrobial stewardship: We need not only to know what to *do* to maintain antimicrobial effectiveness, but to know *why* [29, 30]. The belief that national guidelines negatively impact clinical autonomy may conflict with the role of steward of medical resources. However, in addition to the doctor and a given patient, the wider community has legitimate interests in how health risks are allocated. Thus, it is important to bring population level normative concepts to light, such as solidarity, reciprocity and the common good. We would highlight the importance of including ethical considerations regarding antimicrobial resistance in guidelines for reasonable use of antibiotics.

### Limitations

There are a few limitations to our study. The results are based on a single case study of guideline development in a specific national context. What constitutes good guidelines will be context-dependent given variations in burden of disease, available resources and access to healthcare [7]. Evidence-informed decision-making about national clinical guidelines requires an awareness and understanding of factors potentially affecting development and implementation of the guidelines in the local setting. Neither development process nor guideline content may be directly adopted from other countries experiences, as the professional, cultural and healthcare contexts of the specific countries differ. Thus, the findings of this study may not be directly transferable to other contexts.

We used a deductive theoretical approach and applied pre-conceived categories in the study. Important factors may have been left out of the analysis.

Further, the interviewees were well-educated subjects that may have wanted to appear consistent, rational and highly knowledgeable, which may have framed their responses. Some of them were key participants in the development process and we cannot rule out the possibility that they may have been biased in their description and assessment of the process.

The sample was small and we do not know to which extent the sample resembles the population of interest. A heated debate in the journal of the Norwegian Medical Association suggests a disagreement among health professionals about recommended therapy for sepsis and the evidence base for the recommendation, and indicates mistrust to the process of how comments were dealt with and incorporated within the guidelines [32]. The debate resulted in a GRADE evaluation for this specific issue. This debate was not reflected in our data material.

We do not know if the new guidelines will change the use of antibiotics in Norwegian hospitals and ultimately better the availability of effective antibiotics. In the vast literature on guideline implementation, several barriers and facilitators to implementation are identified that do not relate to the guidelines themselves [7]. Decisions about guideline use include balancing evidence with professional experience and patient preferences. Time pressure, medicolegal concerns, maintaining relationships with patients, and the interests of the individual patient are found to be important reasons to continuing using antibiotics in settings where evidence for limited effect of antibiotic treatment is established [33]. Clinical practice guidelines risk oversimplifying clinical practice, so that accommodating atypical clinical presentations become difficult and professional autonomy is perceived as threatened [7, 31]. Also, decisions are made within a range of constraints, such as the organizational leadership and support and availability of resources, and various determinants on cultural, contextual and behavioral levels may determine hospital antibiotic use [34].

The view that patients should be more involved in their care has gained much support the last decades and there is a quest for better integration of patient preferences into clinical guidelines [7]. In this study, we did not explore or discuss patient views regarding to guideline development.

## Conclusions

Previous empirical studies have documented that clinical practice guidelines have limited influence on health professionals’ behavior and practice. The literature on barriers and facilitators to guideline implementation is growing. Yet, specific knowledge about guideline features associated with compliance and acceptance among target users is sparse. This case study offers an in-depth look of the process by which Norwegian health authorities developed national guidelines for the use of antibiotics in hospitals. The study has identified how health authorities made deliberate choices to enhance relevance, credibility, applicability, ownership and potential acceptability of guidelines by facilitating broad involvement, a formalized and structured development procedure and openness and transparency in the process. Further, the study indicates how several procedural characteristics were perceived by target users as promoting guideline legitimacy; i) diverse perspectives systematically involved in the process, ii) accessibility and transparency of the rationales for decision making, iii) opportunities for appeals and reconsiderations, and iv) regulative authority.

## Abbreviations

AGREE: Appraisal of Guidelines, Research and Evaluation
AMR: Antimicrobial Resistance
ESBL: Extended Spectrum Beta-Lactamase-resistant bacteria
GRADE: Grading of Recommendations, Assessment, Development and Evaluation
MRSA: Methicillin-Resistant *Staphylococcus aureus*
NORM Surveillance Programme: The Norwegian Resistance Monitoring and Surveillance Programme
PICO: Population, Intervention, Comparison and Outcome
